# The impact of climate change and biodiversity loss on the health of children: An ethical perspective

**DOI:** 10.3389/fpubh.2022.1048317

**Published:** 2023-01-20

**Authors:** Phoebe C. M. Williams, Justin Beardsley, David Isaacs, Anne Preisz, Ben J. Marais

**Affiliations:** ^1^School of Public Health, Faculty of Medicine, The University of Sydney, Sydney, NSW, Australia; ^2^Department of Immunology and Infectious Diseases, Sydney Children's Hospital Network, Sydney, NSW, Australia; ^3^Sydney Infectious Diseases Institute (Sydney ID), The University of Sydney, Darlington, NSW, Australia; ^4^School of Women and Children's Health, The University of NSW School of Women's and Children's Health, Sydney, NSW, Australia; ^5^Clinical Ethics, Sydney Children's Hospital Network, Sydney, NSW, Australia; ^6^Sydney Health Ethics, The University of Sydney, Sydney, NSW, Australia

**Keywords:** climate change, child health, global health, medical ethics, biodiversity loss, health inequity

## Abstract

The reality of human induced climate change is no longer in doubt, but the concerted global action required to address this existential crisis remains inexcusably inert. Together with climate change, biodiversity collapse is increasingly driving the emergence and spread of infectious diseases, the consequences of which are inequitable globally. Climate change is regressive in its nature, with those least responsible for destroying planetary health at greatest risk of suffering the direct and indirect health consequences. Over half a billion of the world's children live in areas vulnerable to extreme weather events. Without immediate action, the health of today's children and future generations will be compromised. We consider the impact of biodiversity collapse on the spread of infectious diseases and outline a duty of care along a continuum of three dimensions of medical ethics. From a medical perspective, the first dimension requires doctors to serve the best interests of their individual patients. The second dimension considers the public health dimension with a focus on disease control and cost-effectiveness. The neglected third dimension considers our mutual obligation to the future health and wellbeing of children and generations to come. Given the adverse impact of our ecological footprint on current and future human health, we have a collective moral obligation to act.

## Introduction

Climate change is now recognized as a global health emergency (1). Yet as the planet warms and extreme weather events increase in frequency, it remains difficult to mobilize concerted efforts to mitigate its worst effects. Children are the innocent victims of our inaction. The United Nations Convention on the Rights of the Child mandates that children should have a right to health and be raised in an environment that promotes their optimal development (2). However, up to half the world's 2.2 billion children are at high risk from the impacts of climate change, and this will only increase in coming decades (1). UNICEF has estimated that by 2030, up to 131,000 excess child deaths will occur each year if mitigation strategies to address climate change are not implemented (2–4).

The effects of climate change on child health incorporate the direct physical effects of acute climate-related events and geographic shifts in infectious diseases, as well as the indirect effects of food insecurity and disrupted social order resulting from scarce resources and climate refugee displacement (1). Currently, 820 million children are regularly exposed to the acute effects of heatwaves and 400 million are frequently exposed to high intensity cyclones, while many more are affected by the prolonged deleterious effects of drought and famine (1).

**Figure 1:**
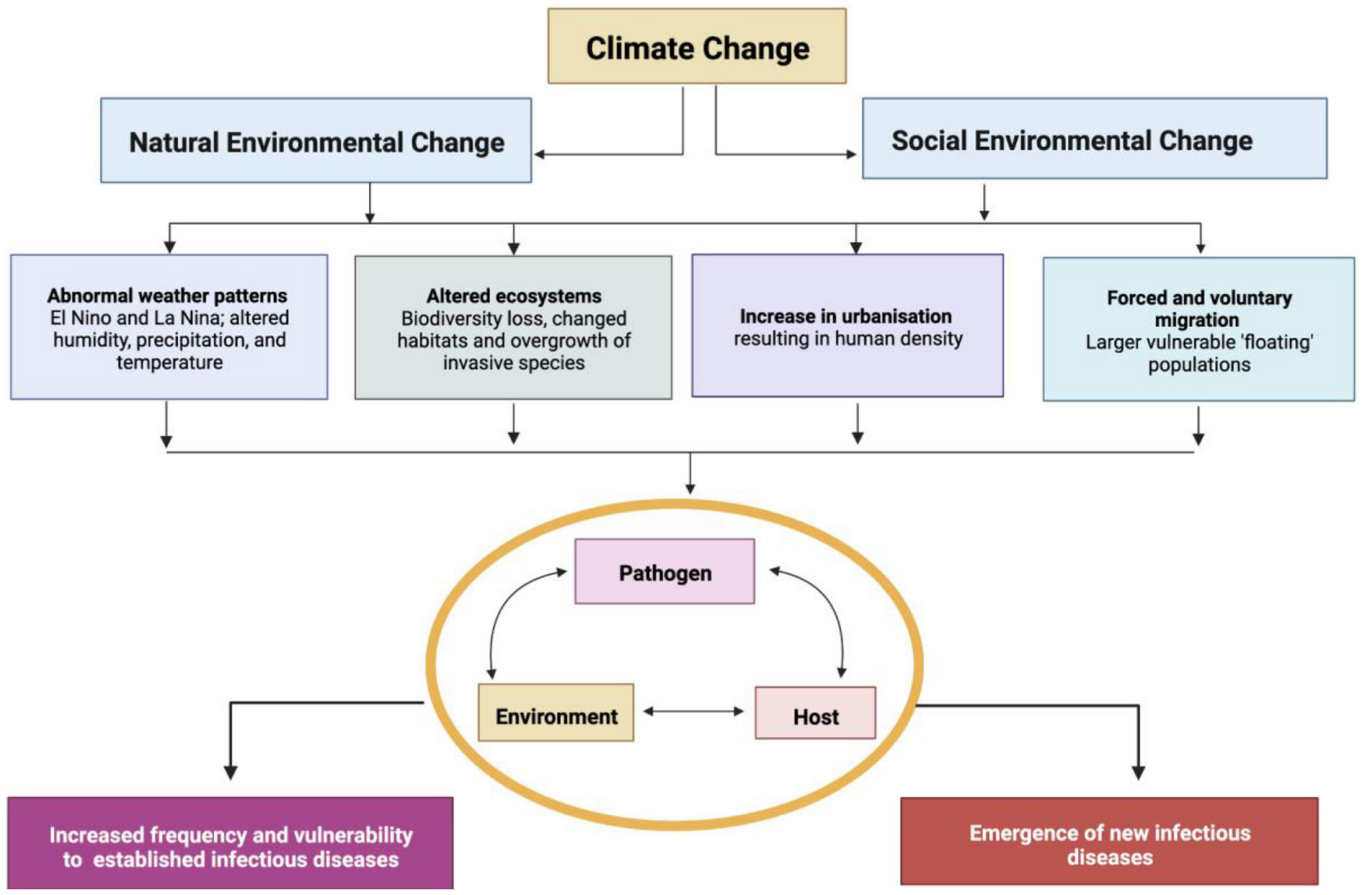
Social and natural environmental changes pose a direct risk to the health of children. Adapted from (9, 10).

For more than a decade the One Health Approach has sought an integrative means to interweave the health of humans, animals and ecosystems, with variable success (5). Our planet's current biodiversity loss has been referred to as the 6th major extinction event, and the first to be driven by human activity (6). Biodiversity loss impacts the functioning and resilience of earth's life-giving ecosystems, whilst diminishing iconic species that have cultural significance (7, 8). Disturbed and fragile ecosystems have especially large impacts on infectious diseases that emerge at the human-animal interface, and those related to environmental degradation or pollution (Figure 1). Children are most prone to the changing ecology of infectious diseases in the face of climate change and biodiversity loss, as the predominant causes of death in children—malnutrition, respiratory tract infections, diarrhea and malaria—will all be impacted by a warming and ecologically degraded planet.

In 2016 the United Nations launched the sustainable development goals, which recognize the interdependence of human health and planetary ecosystems, as well as the biodiversity that sustains them (11). Despite many calls to arms and ever more convincing evidence of human-induced climate change driving poor health outcomes for current and future generations of children, progress has been slow. Our inaction in mitigating climate change may result in today's children, and future generations, being the first to have poorer physical and mental health than generations prior. In this review, we examine intergenerational justice and global health inequities caused by the direct and indirect impacts of climate change and biodiversity loss on child health (2).

## Climate change and global health inequity

The burning of fossil fuels has driven rapid economic development in resource-rich nations, resulting in prolonged life expectancy and strong health systems for their populations. However, following a century marked by massive carbon consumption, the side effects of climate change and environmental degradation present a contemporary ethical quagmire: calls to reduce carbon emissions are coming at a time when developing economics are yet to realize the same health and survival benefits enjoyed by developed economies (12). It is therefore understandable that some low-income nations are reluctant to embrace ambitious climate targets that may limit their growth potential. Yet climate change impacts the entirety of the world's population, and those with limited resources are most at risk of its adverse outcomes (13).

Climate change is regressive in its nature. That is, those most vulnerable to the effects of a warming planet with declining biodiversity are least responsible for creating the problem—including (and exemplified by) children (9). Resource-constrained settings are also least able to mitigate the impacts of global warming, while the world's richest nations—who are responsible for most of the cumulative impact of greenhouse gas emissions—have resources to assist in their response (14). Currently, the 33 countries at highest-risk of the effects of climate change emit <10% of the world's greenhouse gas emissions; and of these 33 countries, 29 exist in fragile contexts (1). None of these highly affected countries have the capability to implement strategies to adapt to the current frequency of environmental shocks or to facilitate a transition to a “green economy” (1).

While global warming is a collective threat driven by highly variable contributions between individuals and societies, global biodiversity is a collective resource that should be protected. Similar to the challenge posed by antimicrobial resistance, biodiversity loss represents a “tragedy of the commons”—whereby a common resource is depleted when self-interest, rather than collective responsibility, prevails (13–15). Both climate change and biodiversity loss are propelled by factors that are inherent to a competitive global environment in which short-term individual or national benefit prevails over the collective good. Consequently, everyone—and especially children and the most vulnerable—are at risk of poor health outcomes that will be amplified in future generations.

**Figure 2:**
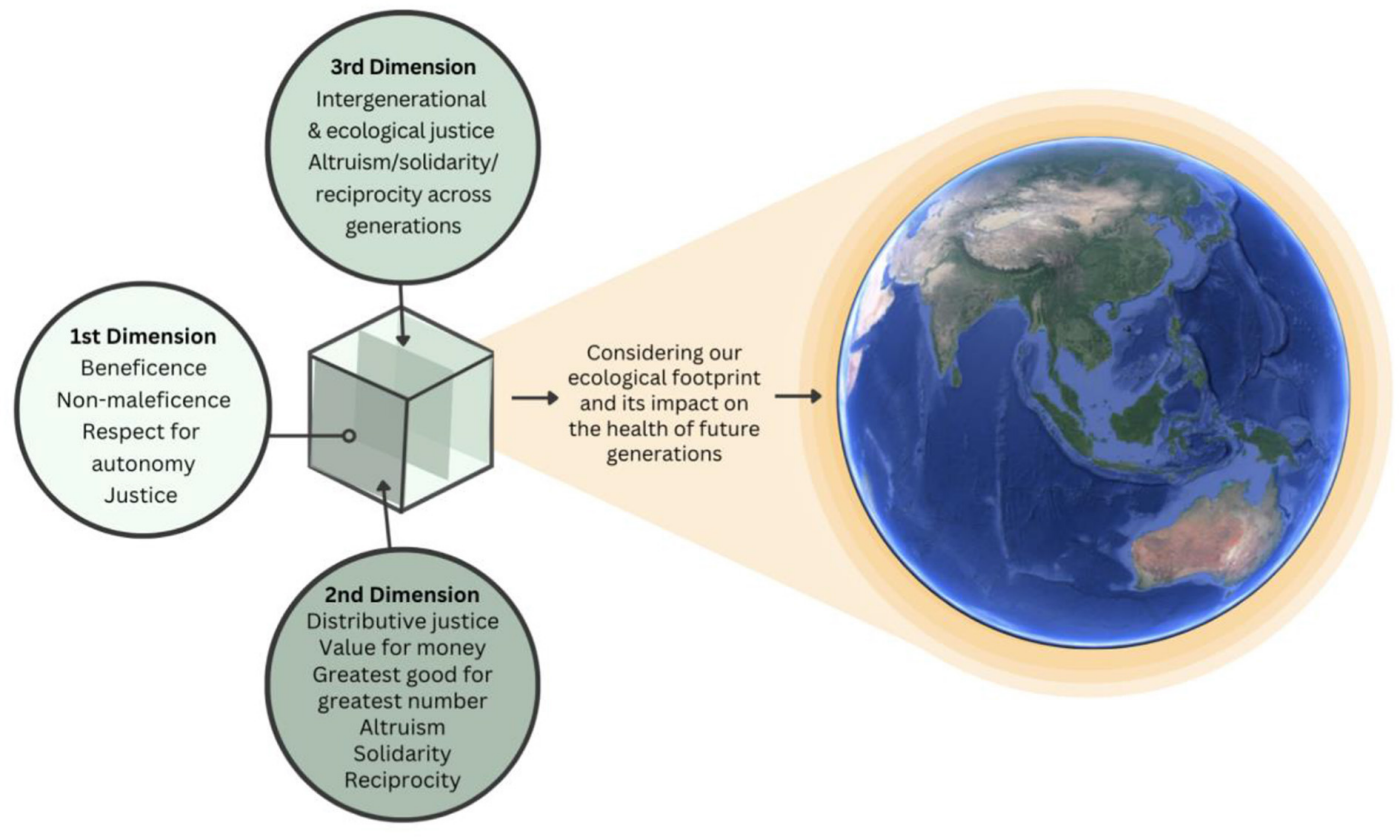
The three dimensions of medical ethics.

Accounting for the rights of unborn future generations in reasoned ethical analysis is challenging, since no existing ethical framework considers true existential threats and ecological tipping points that are within the control of one generation, but mainly impacting on another. As pediatric healthcare professionals, obligations to the health of children in our immediate care are grounded in established ethical frameworks, conceptually proposed as the first dimension of medical ethics. The second dimension of medical ethics emphasizes the need to broaden our view beyond the best interests of the individual patient, by considering broader public health principles and values (16, 17). The concepts of planetary health and intergenerational justice are less established in moral and legal terms; yet our ethical responsibility to also consider the health and wellbeing of future generations has been described as the neglected “third dimension” of medical ethics (Figure 2) (18).

## Climate change impacts on child health

**Figure 3:**
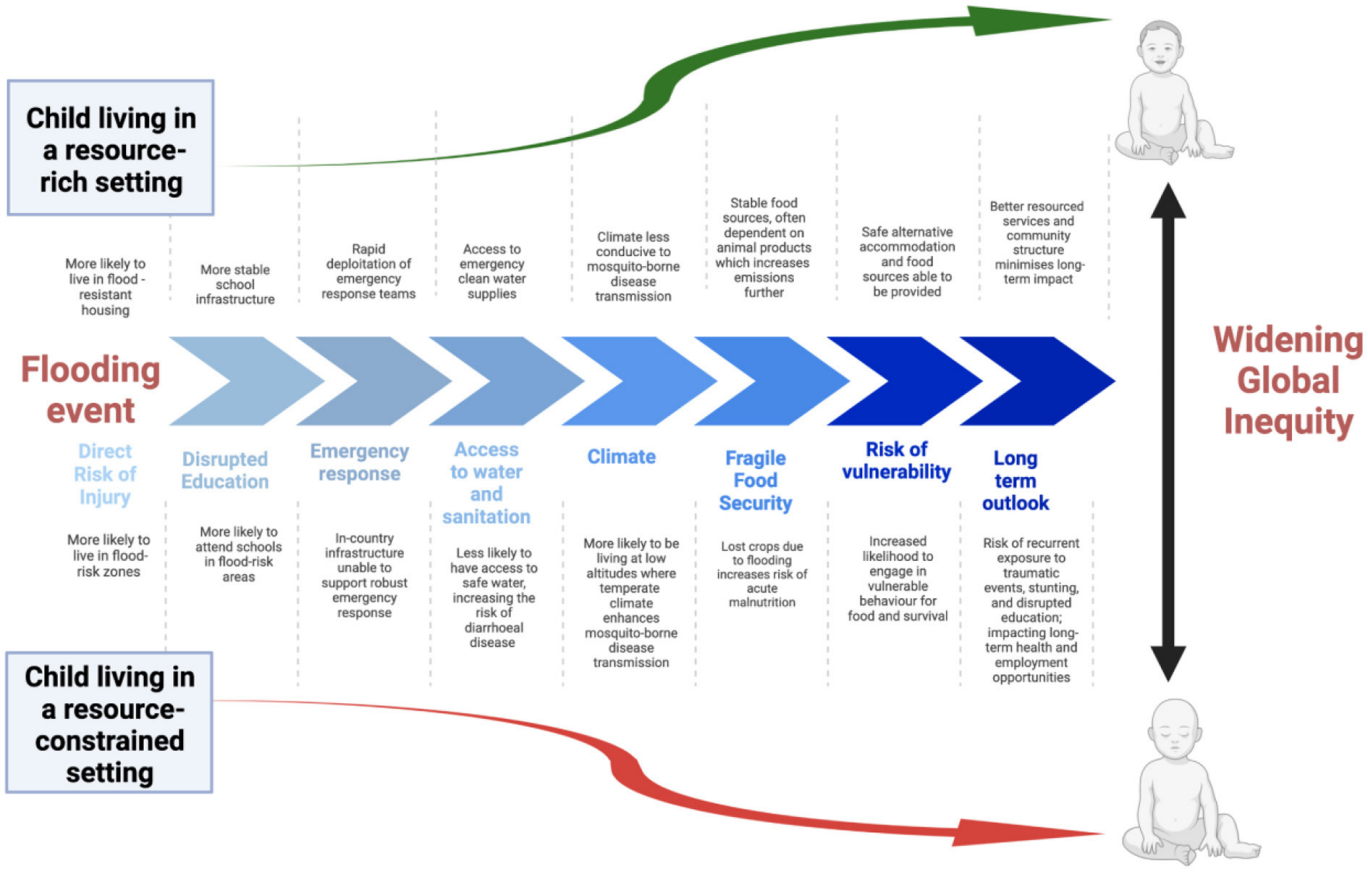
Extreme weather events result in multiple immediate- and long-term impacts on children's health that exacerbate current global inequities, as exemplified by the consequences of a flooding event [modified from (13, 20)].

Most adverse impacts of climate change are concentrated on poorer populations living in low latitudes, where many climate-sensitive disease states are prevalent (9, 19). The most vulnerable regions are those on the fringe of tropical and subtropical settings where infectious disease risks tend to be compounded by under-resourced health and social security systems, especially for people living in informal settlements with high rates of population growth (Figure 3) (21).

At present, 600 million children—over 1 in 4 globally—are frequently exposed to vector-borne diseases, a number which will increase as climatic conditions favorable to mosquitos expand (1). The interaction between global warming, shifting precipitation patterns, and extreme climate events on vector-borne disease patterns is complex. However, an expansion or shift in vector-borne disease distribution results in the introduction of infectious agents into immunologically naïve populations, with considerable health and socioeconomic costs as a consequence (22, 23).

Dengue is the most rapidly spreading mosquito-borne disease worldwide, with its dissemination driven by broad distribution of the *Aedes* mosquito through international trade and travel, followed by swift establishment of local transmission once introduced to non-endemic areas (24, 25). Warming global temperatures are exposing many immunologically naïve populations to dengue disease; as has also occurred with the expansion of malaria, transmitted by the *Anopheles* mosquito species. Both dengue and malaria cause significant morbidity and mortality in children and are spreading into higher altitudes and latitudes. In Nepal, for example, a 1°C increase in mean temperatures has resulted in a 25% higher incidence of malaria, expanding disease transmission from 38 to 65 districts countrywide—including, for the first time, districts located 2,000 m above sea level (26, 27). Similar expansions of malaria into highland regions in Eastern Africa and South America have also been demonstrated (28). Expanding and shifting vector-borne disease distributions threaten to overwhelm existing disease control and surveillance programs, whilst also undermining historical interventions implemented for vector control.

In the Northern hemisphere, tick-borne diseases are demonstrating similar shifts into higher altitudes and latitudes (28, 29). The incidence of Lyme disease is steadily increasing in Europe (30) and North America (31); while Russia is experiencing increasing rates of tick-borne encephalitis as more favorable conditions enable the *Ixodes* tick to thrive (32). Many climate factors also affect the density of rodents, an important zoonotic reservoir for many pathogens (33). A surge in leptospirosis, acquired from rodent-contaminated water through skin abrasions or mucocutaneous membranes, is strongly associated with flooding; whilst climate change has also promoted the spread of multidrug-resistant fungal infections, specifically those due to *Candida auris* (33, 34).

Compromised water and sanitation may also drive infectious disease outbreaks propagated by both episodic climate events and gradual shifts in ecology (35–37). Clean water is already inaccessible to a large portion of the world's population, and those living near major watercourses are at particular risk of water-borne infections (38, 39). Currently, every year over half a million children die from diarrhoeal diseases, whilst 330 million children are frequently exposed to riverine flooding, a number which will rise as glaciers melt and precipitation increases (37, 40). A further 240 million children are exposed to coastal flooding (1). Modeling data suggests that climate change will drive an increase in gastrointestinal infections, such as cholera and *Salmonella* typhi (the cause of Typhoid or enteric fever), due to water source contamination resulting from increased soil erosion and sediment run-off (41–43).

As water becomes more contaminated, clean drinking water will become scarcer. It is estimated that 920 million children—over one-third of children globally—are already exposed to water scarcity, and this number will increase in coming years as the frequency and severity of droughts and water contamination events rise, alongside increasing competition for water (1). Enhanced holistic research approaches to waterborne disease that acknowledge the complexity of these problems and seek lasting ecological solutions are urgently required (44).

Other direct health impacts of climate change on children include the effect of heat waves, which are particularly dangerous for young infants who suffer excess mortality during extremely hot weather (45, 46). Prolonged allergy seasons driven by warmer weather and increased pollen counts may cause morbidity in older children with allergies, while wildfire smoke and other air pollutants are responsible for worsening lung health in children (47, 48). Currently, 2 billion children—almost 90% of children globally—are exposed to ambient fine particular matter that exceeds 1μ/m^3^. The World Health Organization (WHO) estimates that more than half a million children die every year due to acute lower respiratory infections triggered by exposure to air polluted by excessive fossil fuel use (49).

## Climate change and malnutrition

Although the effects of climate change are hard to predict, it is likely that food scarcity will increase, particularly in areas that are already food insecure (12). For every 1°C increase in temperature, global wheat production is estimated to reduce by 6% (50). When food is scarce, children are often the first in the household to become malnourished (51). A malnourished child is vulnerable to higher rates of morbidity and mortality due to infectious diseases, including those outlined above (52).

Malnutrition currently contributes to at least one-third of global under-5 deaths in children, and it is estimated that by 2030 climate change induced malnutrition may cause an additional 95,000 deaths every year (3, 19). Simultaneously, chronic malnutrition causes stunting which already affects 150 million children worldwide, limiting their learning potential and negatively impacting on future employment opportunities (53–55).

Food insecurity also drives mass migration. In 2011, a widespread drought in the Horn of Africa resulted in significant food insecurity across East Africa, with mass displacement of vulnerable populations resulting in the swelling of Dadaab refugee camp in East Kenya to four times its capacity-−350,000 inhabitants—within just 1 month (56). Overcrowding and inadequate sanitation resulted in outbreaks of multiple infectious diseases including dysentery, cholera and hepatitis E virus (56). Many regions of the world may experience similar scenarios as food insecurity and extreme weather events converge and increase in frequency. These considerations are further amplified by climate change impacts being greatest in some of the most densely populated regions of the world—including coastal South Asia, the Mekong Delta, the Nile River basin, the Pacific Islands, Equatorial Africa and the Pacific Coast of Latin America (19, 20, 47, 57).

## Broader impacts on child health

Alongside the many direct impacts of climate change on children's health, a number of indirect effects also disrupt their health and development. Climate change is already increasing the frequency and severity of extreme weather events that disrupt infrastructure critical to their wellbeing, including access to schools and health facilities (Figure 3) (20). These events also divert resources away from routine child health care, reducing access to antenatal and perinatal care and disrupting routine immunization services (19). Water and food shortages induced by climate change drives internal and international displacement, which may result in mass migrations and regional conflict (47). By 2050, there will be an estimated 200 million climate refugees—many of them women and children, who are most vulnerable to adverse health effects resulting from social disruption and dislocation (19, 47, 57).

For a child to thrive, a stable socioeconomic environment is key. Climate change threatens this stability, fragmenting communities and impacting children's mental health and cognitive outcomes. Previous droughts across East Africa resulted in food crises that threatened children's lives, forcing them to leave school to beg for food or engage in hazardous employment activities to survive—exposing them to violence, exploitation and abuse (20).

Climate change is already affecting the psychological development of children across their lifespan (58). Children and adolescents are particularly vulnerable to the psychological impacts of climate change and biodiversity loss (58). Heatwaves, wildfires, droughts and floods can exacerbate existing mental health problems, while existential threats (worry, anger and frustration about the effect of climate change) can aggravate pre-existing symptoms or contribute to the onset of new mental health disorders (59). Extreme weather events may also create trauma through loss or separation from caregivers (19, 47, 60), resulting in rising rates of post-traumatic stress disorder, social phobia and psychotic disorders (61–63), highlighting the importance of urgent mitigation strategies (59).

## Climate change and the third dimension of medical ethics

The interconnectedness between human health and the health of the ecosystems that sustain all life on earth have been acknowledged by bioethicists for decades (64), yet ecosystem collapse and biodiversity loss have not be recognized as major existential threats. Western enlightenment emphasized material advancement, scientific knowledge and technological mastery as virtues, but without adequately considering its environmental impacts (65). The United Nations General Assembly has declared access to a clean and healthy environment a universal human right (66): a right that is severely threatened by climate change and biodiversity loss. Our rapidly declining planetary health should prompt a critical re-evaluation of our medical ethical frameworks to include major existential threats to the health and wellbeing of both current and future generations of children.

Healthcare traditionally emphasizes the first dimension of medical ethics: the doctor-patient relationship, which focuses on the principles of non-malevolence, beneficence, respect for autonomy and justice (18). The second dimension of medical ethics extends into the public health sphere, with consideration of the “greater good,” distributive justice and other utilitarian concepts such as solidarity, reciprocity and altruism. The second-dimension exposes global inequities, particularly the regressive effects of climate change—whereby those most vulnerable and worst impacted are least likely to have driven the deterioration of our planetary health (9). However, our obligation to future generations should also consider intergenerational and reparative justice, altruism, and ecological justice—that is, recognizing the role of earth's life-sustaining ecosystems for the wellbeing of humankind. The third dimension of medical ethics extends our moral obligation as healthcare providers to advocate for the interests of future generations who will inherit our ecological footprint, given the interconnectedness that exists between human actions today and the destruction of our planet's life-sustaining ecosystems (Figure 2) (18, 67). The ethical work of justifying our obligations to future generations of children is important and evolving, and while not fully examined in this paper, this will necessitate developing inter-generational justice frameworks that sufficiently recognize the importance of sustainable ecosystems, to allow all species to flourish on earth now and into the future.

## Conclusion

Climate change is a “wicked problem” that is challenging to address effectively (68); as is balancing our obligation to current and future generations of children. There is a need and urgency to identify and implement actionable solutions (2, 69). Major steps across interlinked policies and programmes could improve both climate and child health, but these will require strong commitment and global collaboration. A focus on our generation's ethical responsibility to protect our own children, as well as future generations of children, is important to help to build the consensus needed for action (70).

The impacts of climate change are cumulative in their nature, and require consideration of many direct and indirect impacts distributed unequally within and between populations and generations (3). Without timely action, the ongoing damage inflicted may be irreversible. Global inequality will be exacerbated as we push against the ecological ceilings of sustainability, and potentially beyond safe spaces for humanity to live and thrive (10, 71–73). The third dimension of medical ethics defines our obligation to protect the health of current and future generations of children, ensuring we preserve the planet's life giving ecosystems as a moral duty.

## Author contributions

BM conceived the article. PW authored the first draft. JB, DI, and AP provided helpful critical feedback which was incorporated into future drafts. All authors contributed to the article and approved the submitted version.
